# Autoimmune Progesterone Dermatitis: A Case Report

**DOI:** 10.1155/2012/757854

**Published:** 2012-08-09

**Authors:** Rachana George, Shawky Z. A. Badawy

**Affiliations:** Department of Obstetrics and Gynecology, Upstate Medical University, Syracuse, NY 13210, USA

## Abstract

*Background*. Autoimmune progesterone dermatitis is a rare cyclic premenstrual allergic reaction to progesterone produced during the luteal phase of a woman's menstrual cycle. Patients present with a variety of conditions including erythema multiforme, eczema, urticaria, angioedema, and progesterone-induced anaphylaxis. *Case*. Thirty-eight-year-old woman G2P2002 presents with erythema multiforme and urticarial rash one week prior to her menses starting one year after menarche. She was treated with oral contraceptive pills and the symptoms resolved. *Conclusion*. This is a typical case of progesterone autoimmunity. The diagnosis is based on cyclic nature of the dermatitis. This differentiates the condition from other allergies or systemic diseases with skin manifestations. Inhibition of ovulation in such cases results in decrease in progesterone secretion and prevention of symptoms.

## 1. Introduction

Autoimmune progesterone dermatitis is a rare disease caused by an autoimmune response to endogenous progesterone in women during their reproductive years [1]. Skin lesions typically occur due to increases in progesterone during the luteal phase of the menstrual cycle [2]. It was first described in 1921 [1]. 

## 2. Case Report

A thirty-eight-year-old married Caucasian female was referred to our reproductive endocrinology clinic by her gynecologist secondary to cyclical urticaria. Patient has a gynecologic history of menarche at the age of 12 and reports 28 day menstrual cycles. Patient states she gets an erythema multiforme, urticaria, and dermatographism one week prior to menses that resolve after menses since one year after menarche. Patient reports no history of joint swelling. Patient as an adolescent was seen by an allergy specialist and dermatologists for evaluation. Allergy testing was done and was reported negative. Patient was placed on antihistamines for treatment, which she reports did not help her symptoms. Patient was told her symptoms were due to stress.

The patient's obstetrical history consists of two pregnancies resulting in two full-term cesarean deliveries. She has not had any issues with infertility. The patient reports during pregnancy and while breast feeding that she did not have any episodes of a rash and/or hives. Patient also states that she was on oral contraceptives for one year at age 23 where she noticed her symptoms decreased. She reports multiple drug allergies resulting in a rash.

Our patient was diagnosed with autoimmune progesterone dermatitis based on history and physical exam and was treated with oral contraceptives which was successful in controlling her outbreaks.

## 3. Comments

Autoimmune progesterone dermatitis is a rare condition that occurs during the second half of the menstrual cycle. Dermatological symptoms include urticaria, eczema, angioedema, deep gyrate lesions, papulovesicular lesions, targetoid lesions, or anaphylaxis. Symptoms usually occur three to ten days prior to the onset of menstrual flow and resolve 2 days into menses [5]. Symptoms of autoimmune progesterone dermatitis correlate with progesterone levels during the luteal phase of the menstrual cycle [3, 4]. Patient clinical presentation can vary in severity from mild urticaria to anaphylaxis [5, 6]. 

The onset of autoimmune progesterone dermatitis is variable. The earliest age reported has been at menarche [7]. Diagnosis is made with appropriate clinical history as well as confirmation with intradermal progesterone skin injections [6]. An aqueous suspension or aqueous alcohol solution of progesterone is the preferred agent of testing as it is less likely to cause an irritant reaction compared to progesterone in oil [6]. The skin reaction may be seen immediate within half an hour or delayed upto 48 hours.

There are some disorders similar to autoimmune progesterone dermatitis. One in particular that should be considered when evaluating a patient is mastocytosis. Mastocytosis is a group of disorders characterized by excessive mast cell accumulation in one or multiple tissues. It is subdivided into two groups. One group is cutaneous mastocytosis which is limited to the skin. The second group is systemic mastocytosis where mast cells infiltrate extracutaneous organs. The typical presentation for mastocytosis is pruritus, epigastric pain, nausea, vomiting, chronic diarrhea, and arthalgias. It can also present at neuropsychiatric changes such as irritability, depression, and mood liability. Patients may also have hepatic and splenic infiltration that may cause portal hypertension resulting in ascites. One cutaneous manifestation of mastocytosis occurs when the patient strokes or rubs their skin and it causes urticaria and erythema around lesions known as Darier's sign. Systemic symptoms can occur in any form but the most common is flushing. The most dramatic symptom is anaphylaxis with syncope or even shock. The symptoms of mastocytosis are not cyclic.

Pregnancy can impact the symptomatology of autoimmune progesterone dermatitis. The increase of maternal progesterone during pregnancy may improve the disease process. Our patient experienced resolution of symptoms during pregnancy. The gradual rise of progesterone during pregnancy may act as a desensitizing agent, ultimately reducing symptoms. Due to increased production of anti-inflammatory glucocorticoids during pregnancy, the decrease in maternal immune response may also play a role in improving symptoms. In addition, autoimmune progesterone dermatitis during pregnancy has been associated with spontaneous abortions [1, 6, 8]. Wojnarowska reported as case of a women who became pregnant, developed erythema multiforme at five weeks of gestation, and had a spontaneous abortion at 10 weeks [8]. 

Treatment of autoimmune progesterone dermatitis is achieved mainly through suppressing ovulation. The first line of therapy is combined oral contraceptives. The use of GnRH agonists has been reported successful in treatment [6]. Another therapeutic agent used to suppress ovulation and improve symptoms is tamoxifen [6]. Danazol has also been reported successful in treating autoimmune progesterone dermatitis [7]. Autoimmune progesterone dermatitis has been shown to be resistant to antihistamine therapy, as displayed in our patient [6]. For patients who experience unremitting symptoms after medical management, bilateral oophorectomy is recommended [9].
